# Highly Branched Bio-Based Unsaturated Polyesters by Enzymatic Polymerization

**DOI:** 10.3390/polym8100363

**Published:** 2016-10-14

**Authors:** Hiep Dinh Nguyen, David Löf, Søren Hvilsted, Anders Egede Daugaard

**Affiliations:** 1 Danish Polymer Centre, Department of Chemical and Biochemical Engineering, Technical University of Denmark, DK-2800 Kgs. Lyngby, Denmark; ndinhhiep@gmail.com (H.D.N.); sh@kt.dtu.dk (S.H.); 2 Hempel A/S, Lundtoftegårdsvej 91, DK-2800 Kgs. Lyngby, Denmark; DAVL@hempel.com

**Keywords:** enzymatic synthesis, one pot synthesis, highly branched polyesters, pentaerythritol, crosslinkable polyesters, controlled glass transition temperature

## Abstract

A one-pot, enzyme-catalyzed bulk polymerization method for direct production of highly branched polyesters has been developed as an alternative to currently used industrial procedures. Bio-based feed components in the form of glycerol, pentaerythritol, azelaic acid, and tall oil fatty acid (TOFA) were polymerized using an immobilized *Candida antarctica* lipase B (CALB) and the potential for an enzymatic synthesis of alkyds was investigated. The developed method enables the use of both glycerol and also pentaerythritol (for the first time) as the alcohol source and was found to be very robust. This allows simple variations in the molar mass and structure of the polyester without premature gelation, thus enabling easy tailoring of the branched polyester structure. The postpolymerization crosslinking of the polyesters illustrates their potential as binders in alkyds. The formed films had good UV stability, very high water contact angles of up to 141° and a glass transition temperature that could be controlled through the feed composition.

## 1. Introduction

For many years, wood has been widely used for outdoor applications. Without protection, wooden structures degrade through various different processes, such as exposure to UV light, moisture, and microorganisms, leading to severe deterioration in properties [1,2]. The most widely used coating system for exterior wood applications is based on alkyds. Because of the versatility of alkyds, their ease of use, and economic attractiveness, around one million tons of alkyds are produced worldwide annually [3]. The alkyd binder is the basis of this system, and its role involves forming a tough and continuous film that is able to expand and contract in response to outside temperatures and moisture, thereby continuously protecting the wooden surface from the environment. An alkyd binder can be considered a hyperbranched unsaturated polyester prepared from a ternary mixture of polyhydric alcohols, bifunctional acids and fatty acids, where the unsaturation provides the drying properties to the final coating formulation [4]. The traditionally used polyol is either glycerol or pentaerythritol or a mixture of these two, which is combined with dibasic acids, such as phthalic acid or isophthalic acid, and various fatty acids [3,5]. Pentaerythritol is more photo-stable than glycerol due to the lack of secondary carbons in its structure [6], which makes this a very favorable component for binders used in exterior coatings that are under constant attack by solar radiation. Considering the movement towards bio-based products, a traditional alkyd formulation already contains a number of bio-based components such as bio-based fatty acids and glycerol. In addition, pentaerythritol has also recently been made available industrially as a bio-based feed material [7]. The challenge, however, lies in replacing petrochemically derived phthalic acid or other popular aromatic diacids which account for up to 50 wt % of alkyds’ composition. Therefore, the current movement towards fully bio-based alkyds is focused on replacing the acids with renewable alternatives.

Two classical methods are used for the industrial synthesis of alkyds, namely the monoglyceride and fatty acid processes [3,5,8]. Both of these processes are carried out by heating a complex mixture at temperatures above 200 °C to obtain products with broad molar mass distributions. In both cases, stoichiometry is usually fixed on a hydroxyl excess, to keep the reaction mass from gelling during production [5]. From an environmental perspective, the main challenge with these two conventional synthetic processes is their high consumption of energy, limitations in terms of renewable monomers (that are often multifunctional and more sensitive) as well as side reactions, including the formation of ethers [5], volatile aldehydes as well as dimers and oligomers [5] that sometimes lead to undesired gelation and thereby loss of product. The various possible side reactions result in a low degree of control over product structures, and it has been shown that alkyds of the same feed composition, albeit synthesized by different processes, may have significantly different coating properties [5,8].

Lipases have long been an interesting topic as a greener alternative for the synthesis of polyesters, as shown in many reviews since the 1990s [9,10,11,12,13,14,15,16]. The most commonly used lipase is *Candida antarctica* lipase B (CALB) [17], for which catalytic activity has been shown through many previous investigations [18,19,20,21,22,23,24,25,26]. CALB is a very robust protein, stable in aqueous media in the pH range from 3.5 to 9.5, able to accept various acyl donors [11], and able to catalyze polycondensations at temperatures down to 50 °C [27]. This enables the polymerization of more reactive monomers such as itaconic acid, carrying a lateral vinyl group, as shown recently for preparation of linear polyesters [28,29,30]. Since catalyzed CALB polymerization can be carried out at much lower reaction temperatures compared to the currently used methods for alkyd synthesis, it is a promising candidate for the greener production of alkyds.

There are currently only few examples of investigations into bulk polyester synthesis from ternary feed compositions of glycerol, dicarboxylic acids, and a monocarboxylic acids [31,32,33]. A common denominator for these investigations has been that they were either based on activated esters, which are currently not available on a full-scale basis, or they included other components such as antioxidants in the investigation, which would prevent future curing as required for application of these unsaturated branched polyesters (UBP) as alkyds. In the present investigation we aim at developing a CALB catalysed one pot bulk polymerization method as an alternative to classical procedures used in alkyd binder synthesis. The method was tested by preparation and characterization of a fully bio-based unsaturated polyester prepared from azelaic acid [34] in combination with glycerol, pentaerythritol and TOFA.

## 2. Materials and Methods

All chemicals were acquired from Sigma-Aldrich (Brøndby, Denmark) and used as received unless otherwise specified. Glycerol was provided from Emmelev A/S (Otterup, Denmark), Tall oil fatty acid (TOFA) and pentaerythritol were from PPG Industries (Søborg, Denmark). TOFA composition was identified by methylation using the method according to Bravi et al. [35] following by subsequent analysis by GC/MS using the NIST 2.0 f database. Azelaic acid was purchased from Sigma-Aldrich and ACROS Organics (Morris Plains, NJ, USA). Novozyme-435 (N435) was supplied by Novozymes A/S (Bagsværd, Denmark). The commercial petroleum-based alkyd binders used was obtained from PPG Industries (Søborg, Denmark). SEC analyses were performed in THF on a Viscotek GPCmax autosampler equipped with a Viscotek TriSEC model 302 triple detector array (RI detector, viscometer detector and light scattering detectors measuring at 90° and 7° (Malvern, Malvern, UK) and a Knauer K-2501 UV detector on two Polymer Laboratories PLgel MIXED-D columns (Polymer Laboratories, Church Stretton, UK). The samples were run at a flow rate of 1 mL/min at 35 °C and molar masses were determined using a calibration based on narrow polystyrene (PS) standards. NMR spectra were recorded using a Bruker Avance II (500 MHz (^1^H) and 125 MHz (^13^C)) or a Bruker Avance (400 MHz (^1^H) and 100 MHz (^13^C)) or a Bruker Avance (300 MHz (^1^H) and 75 MHz (^13^C)) (Spectrospin & Bruker, Rheinstetten, Germany). For ^13^C quantification the spectra were recorded using 1D sequence with inverse gated decoupling with a Bruker Avance II (125 MHz (^13^C)). The instrument parameters were as follows: acquisition time 1 s, temperature 299 °K, relaxation delay 5 s, spectral width 32,895 Hz, 7168 scans. Infrared spectra were measured using a Nicolet™ iS™50 FT-IR Spectrometer from Thermo Scientific (Madison, WI, USA). Differential scanning calorimetry (DSC) was performed on a DSCQ1000 from TA Instruments (New Castle, DE, USA). The thermal analyses were performed at a heating and cooling rate of 10 °K/min. Glass transition temperatures (*T*_g_’s) were measured at the inflection point. Thermogravimetric analysis (TGA) was performed on a TGA Q500 from TA Instruments from 25 to 800 °C at 10 K/min under nitrogen flow. Water contact angle (WCA) measurements were conducted on a Dataphysics OCA 20 (Dataphysics, Filterstadt, Germany). Initially a drop of 6 μL was placed on the surface with the needle inside, and the drop was expanded and retracted at a rate of 0.125 μL/s. All WCA reported are an average of three measurements on three different drops on the surface. Acid number measurement was carried out using a 0.01 M KOH solution in MeOH as the titrant and a solution of phenolphthalein in MeOH as the indicator. QUV test procedure: the dried coating was tested in a “QUV/SPRAY with Solar Eye Irradiance Control” in a test chamber model Q-Lab (Q-lab, Westlake, OH, USA) following the standard method EN 927-6:2006 (E).

### 2.1. Experimental Procedure

#### 2.1.1. General Synthesis Procedure for the Lipase-Catalyzed Preparation of the UBPs

All feed components were added to a round bottomed flask together with a magnetic stirring bar. Three vacuum cycles (evacuation and backfilling with nitrogen) was applied, and after that the reaction mass was heated up to 110 °C under nitrogen and kept there for one hour before the temperature was reduced to 90 °C during one hour. Then, immobilized CALB (N435, 10% by weight, pre-dried using a vacuum oven at room temperature for approximately 24 h) was added to the reaction mass. Three vacuum cycles were performed and then the reaction mass was heated under nitrogen for another 2 h. The pressure was reduced to 4 mbar and the reaction was conducted for typically 25–84 h. The reaction mixture was then dissolved in acetone and N435 was removed by filtration. The solution was then rotary evaporated to dryness and fully dried overnight using a vacuum oven at room temperature and used without further purification.

#### 2.1.2. CALB-Catalyzed Bulk One-Pot UBP Synthesis for Preparation of UBP3

Azelaic acid (2.58 g, 0.014 mol), glycerol (1.26 g, 0.014 mol), Tall oil fatty acid (TOFA) (2.23 g, 0.008 mol) and a magnetic bar were transferred into a 100 mL flask together with a magnetic stirring bar. Three vacuum cycles (evacuation and backfilling with nitrogen) were applied, and after that the reaction mass was heated up to 110 °C under nitrogen and kept there for one hour where reaction mass becomes a clear liquid. Then reaction temperature was reduced to 90 °C during one hour where reaction mass became biphasic with tiny particles. N435 (around 10% by weight), pre dried in a vacuum oven (3 mbar, room temperature, 26 h) was then transferred to the flask. Three vacuum cycles were performed and then the reaction mass was heated under nitrogen for another 2 h. After 2 h, vacuum (4 mbar) was applied to remove water off reaction mass. Reaction was stopped after 25 h and the reaction mixture was then dissolved in acetone (50 mL) and N435 was removed by filtration. The solution was then rotary evaporated to dryness and fully dried overnight using a vacuum oven at room temperature and used without further purification.

#### 2.1.3. Structural Data of UBPs

Molecular spectroscopic data of the structural data of UBP 3 is provided as an example. NMR signal assignments of UBP 3 were established using a combination of ^1^H, ^13^C-NMR, HSQC, HMBC, and COSY spectroscopy (Figures S1–S3). Other UBPs have similar data.

IR (cm^−1^): 3300–3650 (O–H stretch), 3007 (sp^2^ C–H stretch), 2850–2970 (sp^3^ C–H stretch), 1736 cm^−1^ (C=O stretch), 1457 (CH_2_ bend), 1094–1236 (C–O stretch), 725 (sp^2^ C–H bending and –CH_2_– long chain bend).

δ_H_ (500 MHz, CDCl_3_): 5.36–5.26 (m, =CH–), 5.22 (m, CH–O–CO–R, triglyceride unit), 5.04 (quintet, *J* = 4.8 Hz, CH–O–CO–R, 1,2-diglyceride units), 4.27–4.24 (br m, CH_2_–O–CO–R, glyceride units), 4.14–4.07 (m, CH_2_–O–, glyceride units), 4.03 (m, CH–OH, 1,3-diglyceride unit), 3.88 (quintet, *J* = 5.1 Hz, CH–OH, 1-glycerides moiety), 3.69–3.67 & 3.57–3.53 (m, CH_2_–O–, glyceride units), 2.73 (*t*, *J* = 6.5 Hz, =HC–CH_2_–CH=), 2.32–2.25 (m, CH_2_–COO–), 2.07–1.95 (m, –CH_2_–CH=CH), 1.58–1.57 & 1.33–1.21 (m, –CH_2_–), 0.86–0.82(m, –CH_3_).

δ_C_ (125 MHz, CDCl_3_): 173.8 (–COOH), 173.2, 172.8 (COOR), 130.5–127.7 (HC=CH), 72.1 (CH–O–CO–R, 1,2-diglyceride units), 70.2 (CH–OH, 1-glyceride units), 68.9 (CH–O–CO–R, triglyceride unit), 68.1 (CH–OH, 1,3-diglyceride unit), 65.0, 63.4, 62.1, 61.3, (CH_2_–O–, glyceride units), 34.1–33.9 (CH_2_–CO–O–R), 31.9, 31.5, 29.7–28.7, 24.8–24.7 (–CH_2_–), 27.2–27.0 (CH_2_–CH=), 25.6 (CH=CH–CH_2_–CH=CH), 14.05–14.01 (CH_3_).

#### 2.1.4. General Synthesis Procedure for a Classical Alkyd Binder

Pentaerythritol (76.41 g, 0.56 mol), azelaic acid (98.55 g, 0.52 mol), TOFA (213.00 g, 0.76 mol), glycerol (12.61 g, 0.14 mol) and a magnetic stirring bar were added to the reaction flask. Three vacuum cycles were performed and the mixture was then heated under nitrogen to 170 °C and maintained at this temperature for 2.5 h (acid number (AN) = 63.3). The temperature was then increased to 230 °C for 2.5 h and then cooled down to room temperature and left overnight. The next day (AN = 29.0), the remaining TOFA was added (106.00 g, 0.38 mol) and the temperature was increased to 250 °C and maintained for 3 h (AN after 3 h = 9.7), before it was stopped.

#### 2.1.5. Pentaerythritol Tetraazelate (PTA) Mixture Synthesis

Azelaic acid (24.98 g, 132.72 mmol), and a magnetic stirring bar were transferred into a two necked flask of which one neck was connected with a condensing system. The system was heated under stirring to 120 °C, where the acid melted completely. Pentaerythritol (2.26 g, 16.60 mmol) was then added to the reaction mass and the system was heated up to 180 °C. The reaction was stopped after 24 h and the raw product was used without further purification.

IR (cm^−1^): 3600–2300 (broad O–H stretch), 2950–2850 (sp^3^ C–H stretch), 1736 (C=O stretch-ester), 1694 (C=O stretch-carboxyl), 725 (–CH_2_– long chain bend).

δ_H_ (400 MHz, THF-*d*_8_): 10.54 (broad singlet, –COOH), 4.11 (s, C–CH_2_–O–), 2.30 (*t*, *J* = 7.4 Hz, CH_2_–COOCH_2_), 2.21 (*t*, *J* = 7.4 Hz, –CH_2_–COOH), 1.57 (m, –CH_2_–, azelaic acid moiety), 1.33 (broad singlet, –CH_2_–, azelaic acid/azelate moiety).

δ_C_ (100 MHz, THF-*d*_8_): 174.7 (–COOH), 173.0 (–COO–CH_2_) 62.9 (C–CH_2_–O–); 43.1 (C–CH_2_–O–), 34.5 (CH_2_–COOCH_2_), 34.3 (CH_2_–COOH), 30.1, 30.0, 30.0, 25.9, 25.8 (–CH_2_–, azelaic acid/azelate moiety).

## 3. Results and Discussion

### 3.1. One-Pot Enzymatic Polymerization Procedure for Synthesis of Glycerol Based UBPs

#### 3.1.1. Analysis of the Tall Oil Fatty Acid (TOFA) Content

TOFA is a commonly used fatty acid mixture of oleic acid and linoleic acid, which in turn is a residue from wood pulp manufacture. Since the choice of wood used for pulp production decides the fatty acid distribution of TOFA, the chemical distribution of the TOFA composition was analyzed and shown in the supporting information (Table S1). The majority of the TOFA sample consists of the 9,12-octadecadienoic acid (C18:2) and 9-octadecenoic acid (C18:1), which accounts for approximately 44% and 31% of the whole mixture, respectively. In order to evaluate enzymatic polymerization as an alternative to classical alkyd high-temperature preparation, this TOFA was applied without further purification.

#### 3.1.2. Structural Characterization of Glycerol Based UBPs

**Scheme 1:**
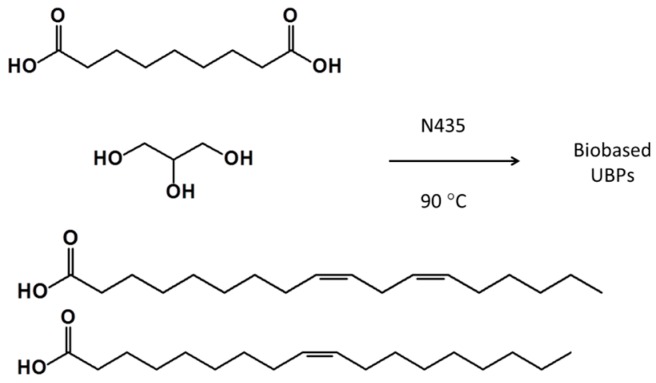
CALB-catalyzed preparation of UBPs by one-pot bulk polymerization between azelaic acid, glycerol, and TOFA.

Bio-based UBPs were synthesized in a one-pot process from TOFA, glycerol and azelaic acid as a renewable alternative to the phthalic acid or anhydride normally applied for alkyd synthesis. The UBPs were prepared directly from the mono and difunctional carboxylic acids without further derivatization, as illustrated in Scheme 1.

The method facilitates the easy preparation of UBPs in a process that can be compared directly with the current industrially applied high-temperature reactions.

**Figure 1:**
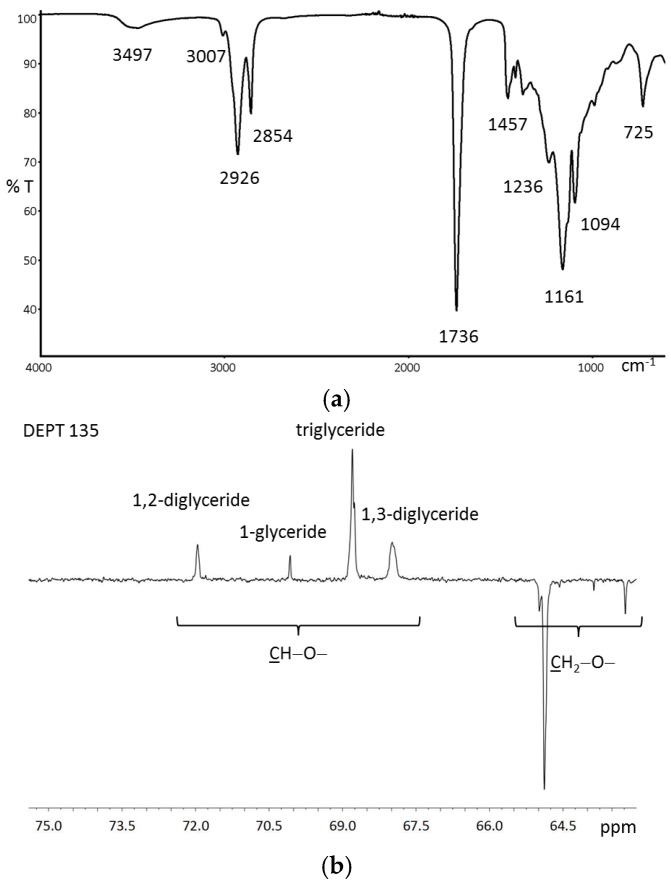
Spectroscopic analysis of the UBP obtained from a feed composition of glycerol, azelaic acid, and TOFA with a molar ratio of 1:1:0.57 (UBP3). (**a**) FT-IR spectrum; (**b**) Expanded DEPT135 NMR spectrum.

As a standard composition glycerol, azelaic acid, and TOFA were reacted in a molar ratio of 1:1:0.57 for 25 h using the general procedure at 90 °C (UBP3—Table 1). This resulted in a product with the expected high conversion of all the mono- and dicarboxylic acids in the feed, as shown by FT-IR in Figure 1a, through a clear absorption band at 1736 cm^−1^ (C=O of unsaturated ester) and excess hydroxyl groups by the O–H stretching at 3497 cm^−1^, as would be expected from a hyperbranched material prepared with a hydroxyl excess.

UBPs prepared from such ternary mixtures can potentially contain up to five different glycerides, and a combination of NMR methods was therefore applied to determine the distribution of glyceride structures in the product. DEPT135, in combination with HSQC, was used to identify the positions of CH groups in the glyceride unit as well as to quantify the content of the various glycerides, as shown in Figure 1b. The DEPT135 spectrum clearly identifies the CH carbons at 67 to 73 ppm, thus enabling quantification from a combination of HSQC and ^1^H-NMR. In the HSQC spectrum the carbons are clearly coupled with their corresponding protons (Figure S4), and a comparison with the literature data on glycerides obtained from esterification between glycerol and monocarboxylic acids permitted assignment of the chemical shifts for the various types of glycerides [36,37,38,39,40,41]. As indicated in Figure 1b, traces of 1-glyceride, 1,2-diglyceride, 1,3-diglyceride, and the triglyceride were identified in the product. Significantly, no 2-glyceride units were determined, which confirms the generally accepted high regio-selectivity of N435 in terms of primary relative to secondary hydroxyl groups [24,42,43,44,45]. The relative intensity of the glyceride unit from a quantitative ^13^C-NMR spectrum was applied to calculate the conversion, which was determined to be 97% of the carboxylic acids (Figure S5).

#### 3.1.3. Testing Possibilities with the Enzymatic Procedure

The robustness and variability of the enzymatic method in terms of feed composition as well as reaction time was investigated systematically to yield UBPs with different structures, molar masses, and physical properties, as shown in Table 1.

The reaction is generally very robust and in none of the cases did the variations in feed composition lead to crosslinking. All of the prepared compositions resulted in molar masses between 20,900 to 39,700 g/mol with polydispersity indexes (PDI) between 3.2 and 5 showing that the prepared polymer are highly branched and that high conversion is achieved during the first 25 h of the polymerization. Changing the acid composition (azelaic acid/TOFA) by decreasing the amount of TOFA in the feed from a molar ratio of 1.13 to 0.29 (UBP 1–4 in Table 1) resulted in an apparent reduction in molar mass, even though it would be expected that reducing the amount of monofunctional acid compared to a difunctional acid in the feed should result in a higher molar mass UBP. Similarly, increasing the amount of diacid in the feed composition from UBP4 to UBP5 also resulted in an apparent reduction in molar mass. This was attributed to the use of a PS-based calibration for molar mass determination, since increasing the degree of polymerization for the UBP results in both higher molar mass as well as increased branching, which will result in a lower hydrodynamic volume of the polymer, and thereby a lower molar mass determined by a PS-calibration.

The changes in the feed composition can also directly be observed in IR, where hydroxyl groups appear when the feed composition changes from hydroxyl deficiency to hydroxyl excess (Figure S6). At high TOFA content in the feed mixture, only triglyceride units were formed (hydroxyl deficiency), whereas the three other glyceride units appeared and increased in intensity as the hydroxyl content was increased, as shown in the ^13^C-NMR stacked spectra of UBP 1–4 (Figure S7).

Longer reaction time (UBP6 compared to UBP4) resulted in the expected increase in molar mass, where a reaction time of 59 h resulted in a molar mass of 39,700 g/mol. The relative intensity of glyceride CH groups in the two products (UBP4 and UBP6), measured by ^13^C-NMR spectroscopy, showed that the 1-glyceride and 1,3 diglyceride units participated in further esterification to yield more triglyceride units (Figure S8). This is expected from a combination of transacylation reactions occurring after approximately 24 h of reaction time, as well as additional esterification reactions bringing the system to an overall higher conversion.

Similarly, the reduction in TOFA content in feed composition also directly resulted in increased glass transition temperatures of the prepared UBPs. At the highest TOFA content the lowest glass transition temperature of −72 °C was observed, whereas this systematically increased to −54 °C for the lowest content of TOFA. This is a result of the number of dangling chains of TOFA leading to a generally highly mobile polymer system with a low glass transition temperature at high content and to a less mobile and more rigid system with a lower content of TOFA, respectively.

All the prepared UBPs were coated onto a cardboard substrate using a standard coating knife and cured into a solid film, by employing a standard cobalt drying system traditionally used for alkyds. Such systems significantly reduce the total drying time by accelerating the crosslink formation rate between alkyd molecules through catalysis of hydroperoxide decomposition [4]. The films were then subjected to DSC analysis and water contact angle measurement (WCA). The cured films clearly show the reverse trend in glass transition temperature with respect to TOFA content compared to the pristine uncured UBPs. With a high content of TOFA there are more cross-linkable groups in the UBP and therefore the fully cured film from UBP1 shows the highest glass transition temperature of −21 °C, whereas the cured film of UBP4 with a low amount of TOFA only has a glass transition temperature of −37 °C. The amount of TOFA in the feed thereby directly can be used to control the flexibility of the cured films.

WCA measurements (Table 1) showed that the acid/diacid composition can be used to control the hydrophilic/hydrophobic balance of the films. The WCAs increased from an advancing contact angle of 104° to 117° by decreasing the TOFA content from 1.13 to 0.27, whereas increasing the content of diacid resulted in a further increase in advancing WCA up to 141° (Figure S9). Compared to the cured classical alkyd prepared from the same materials with a value of 70° ± 1°, the cured UBPs show significantly higher WCAs, which appears as a promising property for novel alkyd systems based on the enzymatic UBPs.

Cured films of UBPs 2 to 6 were subjected to a QUV exposure, where only UBPs 4 and 6 containing the lowest amount of TOFA showed UV durability beyond 350 h of exposure, which is comparable to that of currently used commercial binders.

### 3.2. Increased Degree of Branching by Use of Pentaerythritol

#### 3.2.1. Synthesis of Pentaerythritol Tetraazelate (PTA) Mixture

For enzymatically prepared UBPs to become an alternative to currently prepared alkyd binders it is a requirement that also pentaerythritol can be used as a feed component, since this is well known to give an increased UV stability as well as improved flexibility in coatings. To our knowledge, pentaerythritol has currently not been incorporated into UBPs by enzymatic processes. Pentaerythritol cannot directly be incorporated into the feed mixture as this results in multi-phase mixtures that cannot be polymerized using the enzyme. This is attributed to its low solubility in the reaction mass, which keeps it from interacting with the bonding site on the enzyme channel used for holding the alcohol moiety of CALB [11]. It is well known that pentaerythritol requires derivatization to become soluble in most common solvents, which happens gradually during normal ester synthesis. This is apparently not possible through enzymatic bulk polymerization alone, whereas the high melting point of pentaerythritol (263 °C) is too high to allow oligomerization to take place in the one-pot process.

**Scheme 2:**
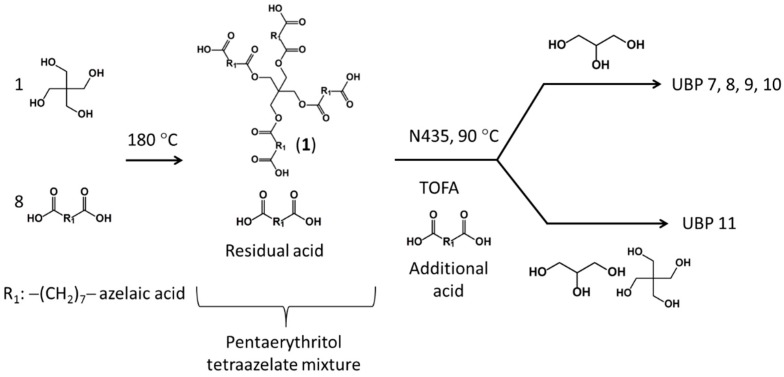
Two-stage synthesis of UBPs containing pentaerythritol. In the first step PTA was prepared from pentaerythritol using an excess of azelaic acid. Subsequently glycerol and TOFA as well as CALB were added and polymerization took place in the second stage.

Inspired by the work of Emelyanov et al. [46], who prepared esters of pentaerythritol by use of a simple melt process, a pretreatment step for preparation of PTA (**1**) was investigated. It was hypothesized that the tetraester would be an alternative to adding the pure pentaerythritol to the enzyme-catalyzed polycondensations as outlined in Scheme 2.

The PTA intermediate is a liquid mixture of excess azelaic acid as well as the tetraester as the main product, which was confirmed by FT-IR, 1D and 2D-NMR (Figure S10a–c). IR analysis of the product showed an absorption band for the carboxyl groups at 1694 cm^−1^ and a much smaller one for the ester at 1736 cm^−1^, due to the much higher content of carboxyl groups (Figure S10a). Methylene groups in the acylated moiety of mono-, di-, tri- and tetraacylated pentaerythritol units can be determined as four separate singlets at 4.05, 4.06, 4.08 and 4.11 ppm in the ^1^H-NMR spectrum [47], whereas in this case only one signal for the tetraacylated units at 4.11 ppm was seen, showing that all of the pentaerythritol hydroxyl groups were acylated (Figure S10b). Partial esterification of the excess carboxyl groups at 174.7 ppm resulted in a smaller signal at the higher field of 173.0 ppm in the ^13^C-NMR spectrum, and subsequent HMBC correlations then helped to define the methylene groups adjacent to these functional groups at 2.21 and 2.30 ppm, respectively (Figure S10c). The ratio of approximately 1:1:3 between the methylene groups of the pentaerythritol moiety and those adjacent to the ester and free carboxylic groups in the ^1^H-NMR spectrum (Figure S10b) confirms that the product mixture comprise mainly PTA and the remaining azelaic acid. Coupling between the pentaerythritol derivatives cannot be fully suppressed since the process is taking place under high concentration conditions, and traces of both the dimer, trimer and tetramer was observed by LC-MS analysis of the crude reaction mixture (Figure S11). This reaction mixture is well-suited for use in preparation of the UBPs, since it is liquid and fully miscible with the other components.

#### 3.2.2. Optimization of Glycerol-Pentaerythritol Based Enzymatic Alkyds

The PTA mixture was employed directly in the established CALB-catalyzed alkyd synthesis procedure together with additional azelaic acid, TOFA and glycerol or a mixture of glycerol and pentaerythritol (Scheme 2). Due to the simplicity in synthesizing the PTA, this procedure enables easy variation in the feed composition for UBPs through the two-stage one-pot process. The molar ratio between azelaic acid and PTA was maintained constantly at 20:1 and the exact functional group ratio between the carboxyl group and hydroxyl group (from glycerol) in UBP4 was used in further development of the two-stage procedure and the feed composition was varied as shown in Table 2.

The complex quaternary feed composition used in the 2-stage process resulted in monomodal SEC traces (Figures S21–S25) from the UBPs illustrating good incorporation of all the components into the polymer. The UBPs were polymerized to high conversion, as is clear from the high *M*_w_ and only one sharp C=O absorption band at 1736 cm^−1^ in the IR spectrum, showing the robustness of the method. The presence of excess OH groups and the unsaturations from the fatty acid were also confirmed by the vibrations at 3464 cm^−1^ and at 3006, 755 and 726 cm^−1^, respectively (Figure S12). All of the prepared UBPs from the two-stage process resulted in polymers with low glass transition temperatures comparable to UBP4 around −55 °C, which shows that the variations in the TOFA content is the main contributor to changes in glass transition temperature.

When the polymerization time was increased (UBPs 7–9, Table 2), the molar mass increased linearly, as would be expected from higher conversion. This was corroborated with the acid number measurements, which were reduced with increasing polymerization time. The polydispersity of the samples also increased with the reaction time illustrating that a more branched molecule was formed at longer polymerization times. Similarly reducing the hydroxyl excess by lowering the glycerol content in the feed mixture resulted in a higher content of free carboxyl groups, as observed with the significant increase in acid numbers (UBP10, Table 2). In the IR spectrum (Figure S13), free carboxyl groups were observed through the broadened carbonyl group at 1736 cm^−1^ and broad O–H stretching vibration from 3400 to 2400 cm^−1^. The stoichiometric mixture resulted in a significant reduction in molar mass compared to the other UBPs. It was also possible to exchange a minor amount of the glycerol for pristine pentaerythritol dissolved in PTA, though this only resulted in minor differences compared to the other UBPs. The amount of pentaerythritol that can be directly added in this fashion is too low to permit exploiting this as a way to increase the pentaerythritol content.

The prepared UBPs were coated onto a glass surface. The prepared coatings show similar WCA as observed for UBP4, though the higher acid content in UBP10 also here results in a high WCA of 13° (Figure S14). The glass transition temperatures after curing of UBPs 7–11 can all directly be compared with UBP4 and are all reduced by approximately 10 °C as a result of the higher branching of the polyester backbone due to the higher pentaerythritol content. All of the enzymatically prepared UBPs show significantly increased WCAs compared to classical alkyd binders with a similar chemical composition, which were observed to have a WCA of around 70°. A brief QUV evaluation of UBP11 showed that this had a UV durability beyond 350 h of exposure. The results from these UBPs in terms of synthetic availability as well as film-forming properties are promising for application of these materials as alkyd binders, and we are currently investigating formulated alkyds based on these materials. In addition to enabling the enzymatic synthesis of polyesters, the stability of the enzymes during repeated use should also be taken into account. Recently there has been a study of the stability and reusability of N435 under different conditions by Poojari et al. [48], where it was seen that specific reaction conditions are required in order to protect the enzyme against particularly mechanical shear. It is a requirement that a stable process can be established, where repeated use of the enzymes is possible, before the process can be implemented commercially.

## 4. Conclusions

An enzyme-catalyzed bulk polymerization method for direct production of UBPs has been developed and evaluated by preparing a number of bio-based UBPs from industrially available raw materials. The developed enzymatic method is simple to perform, robust and allows the preparation of UBPs with much higher control over the chemical structure, preventing unintended side reactions and gelation from occurring during synthesis, compared with the corresponding classical methods currently applied for alkyd binder synthesis. The method was shown to be applicable to a classical feed containing glycerol, TOFA and azelaic acid, and in addition to this a new protocol for the introduction of pentaerythritol into enzymatic polymerizations was also presented. The method permits easy variation in reaction conditions and composition, without any premature gelation, which makes it possible to optimize the UBP structure to a specific degree of branching. The bio-based UBPs show very characteristic high WCA, low glass transition temperatures and good UV stability for coated and cured samples. These are promising results for the future exploitation of these materials as alkyd binders. The method permits easy synthesis of a range of novel alkyds, and it also opens the way for introduction of more sensitive bio-based feed components that cannot survive the high reaction temperatures used in classical alkyd synthesis.

## Figures and Tables

**Table 1:** Molar masses and glass transition temperatures of the pristine UBPs as well as glass transition temperatures and WCA determined for the coated and crosslinked UBPs.

UBP	Molar ratio ^1^		React. time (h)	*M*_w_ (g/mol)	PDI	*T*_g_ (°C)	Film properties	
	Azelaic acid	TOFA					*T*_g_ (°C)	Adv. WCA (°)
1	1	1.13	25	37,400	5	−72	−21	104 ± 1
2	1	0.85	25	27,400	3.6	−66	−27	104 ± 2
3	1	0.57	25	28,100	3.6	−60	−30	109 ± 1
4	1	0.29	25	30,400	3.5	−54	−37	117 ± 2
5	1.16	0.27	25	20,900	3.2	−42	−34	141 ± 1
6	1	0.29	84	39,700	3.8	−52	−35	111 ± 1

^1^ Glycerol molar ratio is constantly kept at 1:1 compared to azelaic acid.

**Table 2:** Molar masses and glass transition temperatures of the UBPs containing PTA, glycerol, TOFA as well as glass transition temperatures and WCA determined for the coated and crosslinked UBPs.

UBP	React. time (h)	*M*_w_ (g/mol)	PDI	*T*_g_ (°C)	Acid number	Film properties	
						*T*_g_ (°C)	Adv. WCA (°)
7	25	34,200	3.3	−51	31.7 ± 0.4	−27	104 ± 2
8	48	40,600	3.4	−51	26.0 ± 0.6	−27	104 ± 2
9	110	56,700	4.5	−55	20.1 ± 0.9	−25	114 ± 3
10 ^1^	25	16,800	2.8	−54	87.3 ± 1.7	−31	132 ± 3
11 ^2^	25	26,800	2.9	−53	28.3 ± 0.6	−28	114 ± 1

^1^ A stoichiometric amount of alcohol and acid was used. ^2^ A mixture of glycerol and pentaerythritol with the molar ratio of 44:1 was used.

## Supplementary Materials

The following are available online at www.mdpi.com/2073-4360/8/10/363/s1, Table S1. Fatty acid (methyl ester) composition of the TOFA sample, Figures S1–S25 with additional NMR spectra, IR spectra, pictures of WCA measurements, LC-MS analysis of PTA and SEC traces of the UBPs.
