# Supplementary material for: Highly Branched Bio-Based Unsaturated Polyesters by Enzymatic Polymerization
Source: Polymers (Basel). 2016 Oct 14;8(10):363. doi: 10.3390/polym8100363 (PMC6432132; doi:10.3390/polym8100363)
Supplement: Supplementary file 1 [file polymers-08-00363-s001.pdf]

Supplementary Materials: Highly Branched Bio-Based Unsaturated Polyesters by Enzymatic Polymerization

Hiep Dinh Nguyen, David Löf, Søren Hvilsted and Anders Egede Daugaard

Table S1. Fatty caid (methyl ester) composition of the TOFA sample.

No.	IUPAC Name	wt %
1	Hexadecanoic acid, methyl ester	0.10
2	Hexadecanoic acid, methyl ester	0.23
3	9-Octadecenoic acid, methyl ester	2.04
4	Octadecanoic acid, methyl ester	0.68
5	9-Octadecenoic acid, methyl ester	31.06
6	Methyl 5,9-octadecadienoate	0.89
7	14,17-Octadecadienoic acid, methyl ester	1.42
8	9,12-Octadecadienoic acid, methyl ester	43.64
9	9,12-Octadecadienoic acid, methyl ester	0.40
10	7,10- Octadecadienoic acid, methyl ester	0.54
11	γ-linolenic acid, methyl ester	6.91
12	Methyl 5,9,12-octadecatrienoate	0.09
13	Ethyl 9,12,15-octadecatrienoic acid, methyl ester	0.07
14	Methyl 9,10-methylene-octadec-9-enoate	0.09
15	Methyl 6-cist,9-cis,11-trans-octadecatrienoic acid, methyl ester	0.93
16	9,12,15-Octadecatrienoic acid, methyl ester	0.70
17	Eicosanoic acid, methyl ester	0.75
18	Methyl 9-cis,11-transs-octadecadienoate	0.47
19	4-(5-pentyl-3 a,4,5,7 a-tetrahydro-4-indanyl) butanoic acid, methyl ester	0.27
20	6,9,12-Octadecatrienoic acid, methyl ester	0.87
21	9,12 Octadecadienoic acid, methyl ester	3.91
22	Methyl 5,9,12-octadecatrienoate	0.22
23	6,9,12-Octadecatrienoic acid, methyl ester	0.61
24	9,12,15-Octadecatrienoic acid, methyl ester	0.60
25	6,9,12-Octadecatrienoic acid, methyl ester	0.36
26	8,11-Eicosadienoic acid, methyl ester	0.25
27	9,12,15-Octadecatrienoic acid, methyl ester	0.30
28	Methyl 5,11,14-eicosatrienoate	0.86
29	Methyl 7,11,14-eicosatrienoate	0.12
Overall		99.37

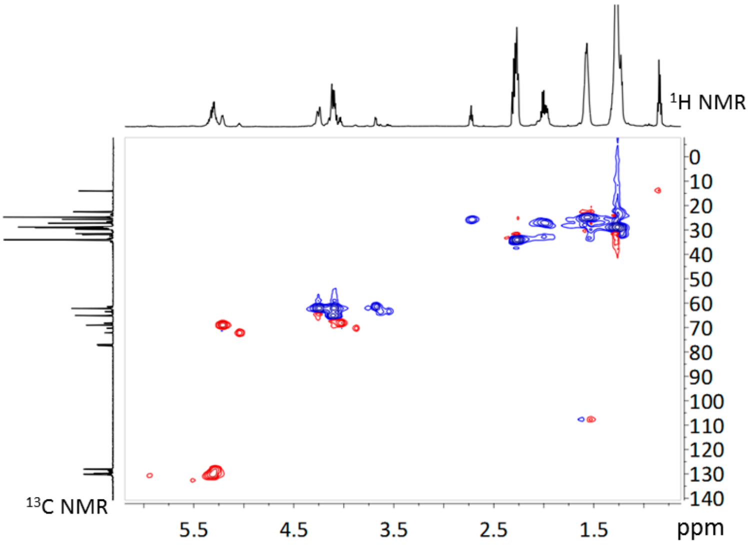

Figure S1. Full HSQC spectrum of UBP3.The **red color** shows the proton-carbon correlations in –CH– and –CH_3 groups while the **blue color** shows the proton-carbon correlations in –CH_2– groups.

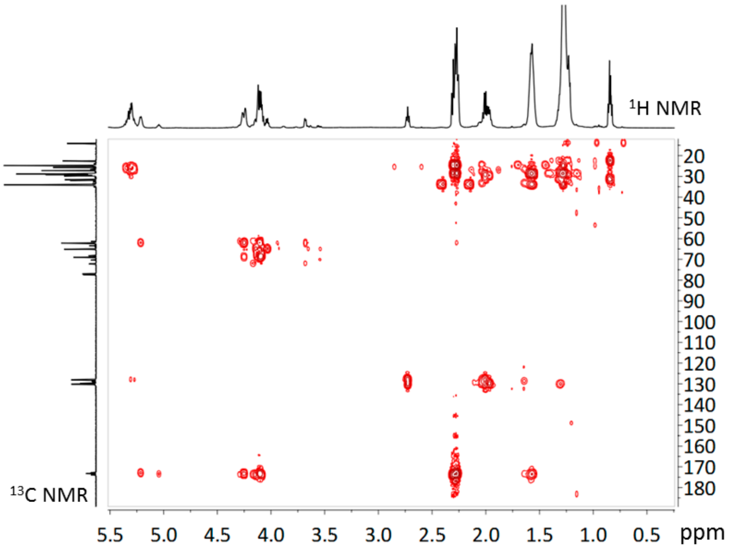

Figure S2. Full HMBC spectrum of UBP3.

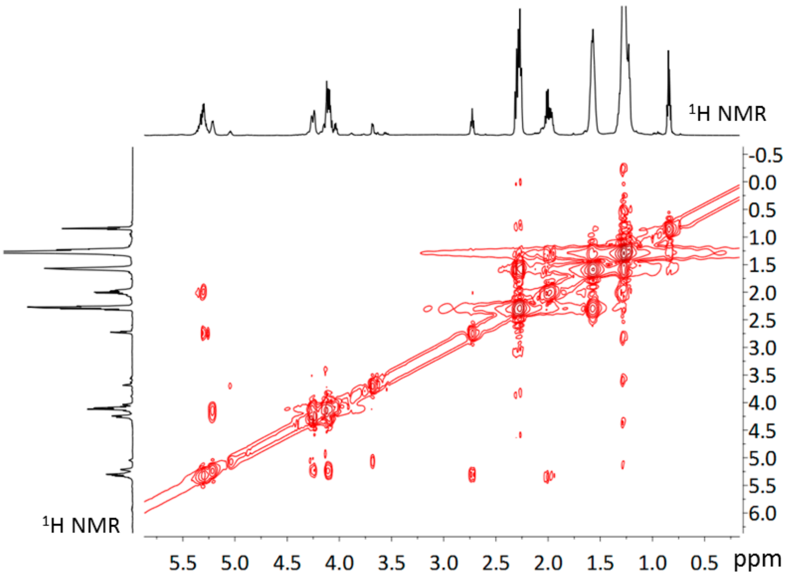

Figure S3. Full COSY spectrum of UBP3.

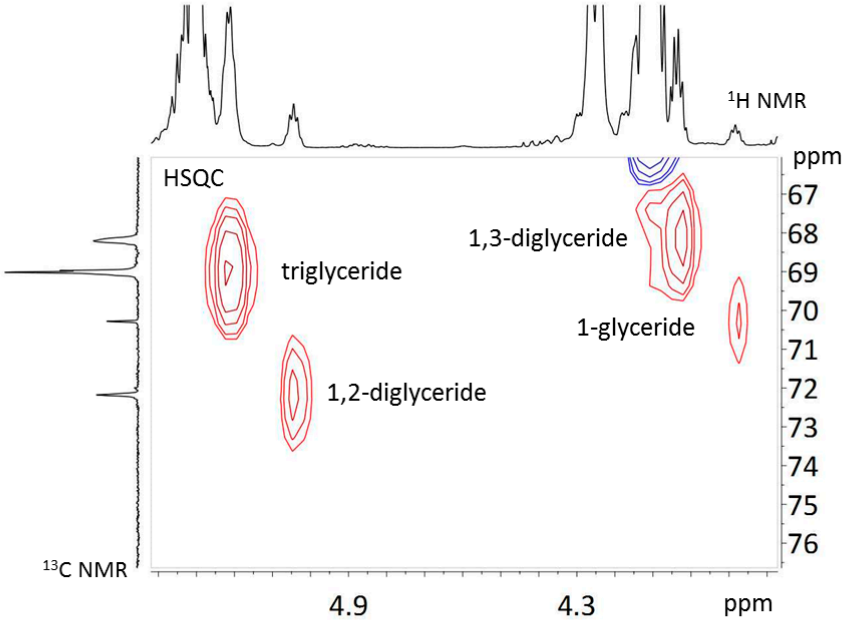

Figure S4. Expanded HSQC spectrum of UBP3 showing correlations for the CH–O groups. The **red color** shows the proton-carbon correlations in –CH– and $–CH_3$ groups while the **blue color** shows the proton-carbon correlations in $–CH_2–$ groups.

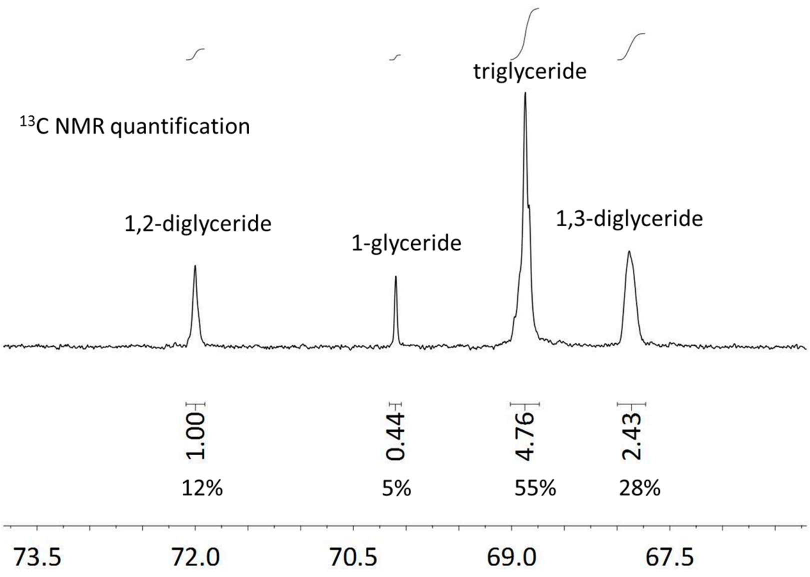

Figure S5. Quantified ^{13}C-NMR spectrum of UBP3.

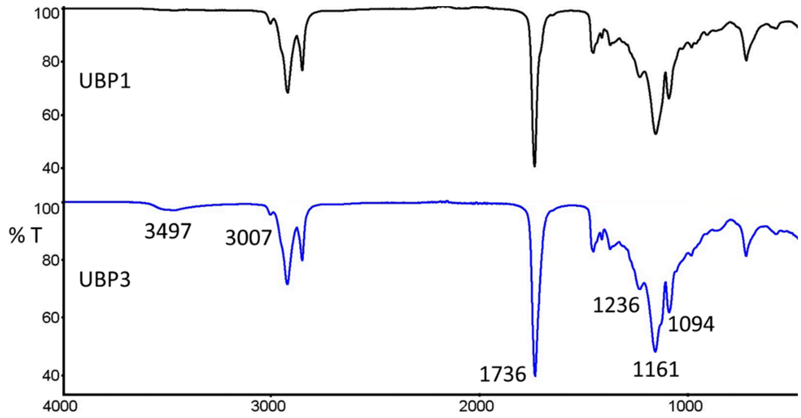

Figure S6. IR stacked IR spectrum of the UBPs obtained from feed composition with an excess (black) and a deficiency (blue) of TOFA.

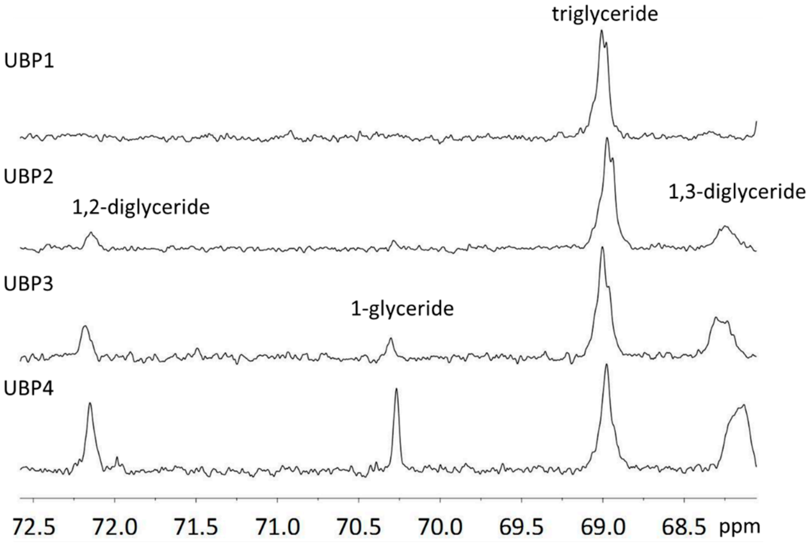

Figure S7. Stacked and expanded ^{13}C-NMR spectrum of the one-pot enzymatic alkyds obtained by decreasing TOFA content.

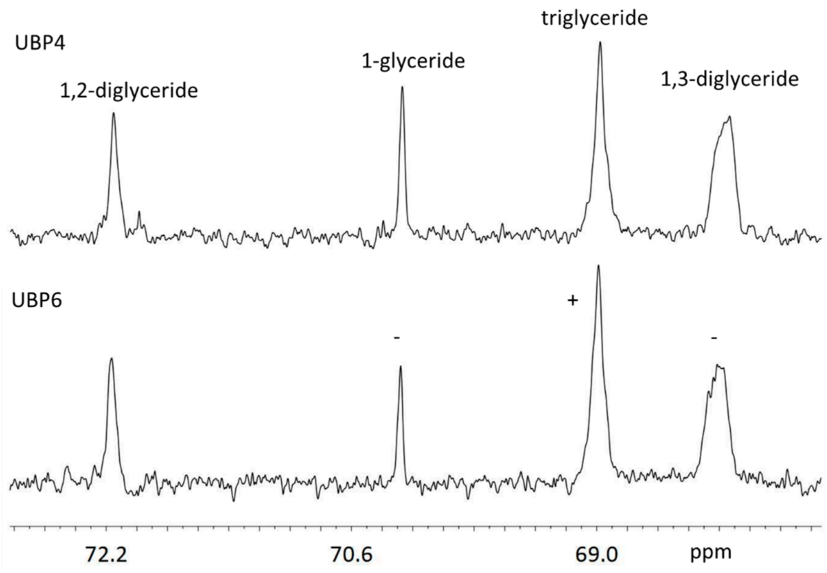

Figure S8. Comparison of ^{13}C-NMR spectra of the product after 25 h (**up**) and 84 h (**down**).

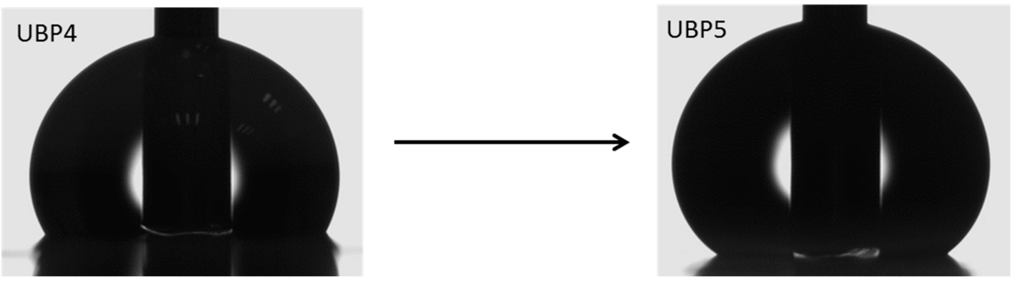

Figure S9. Increase of WCA with increasing diacid content.

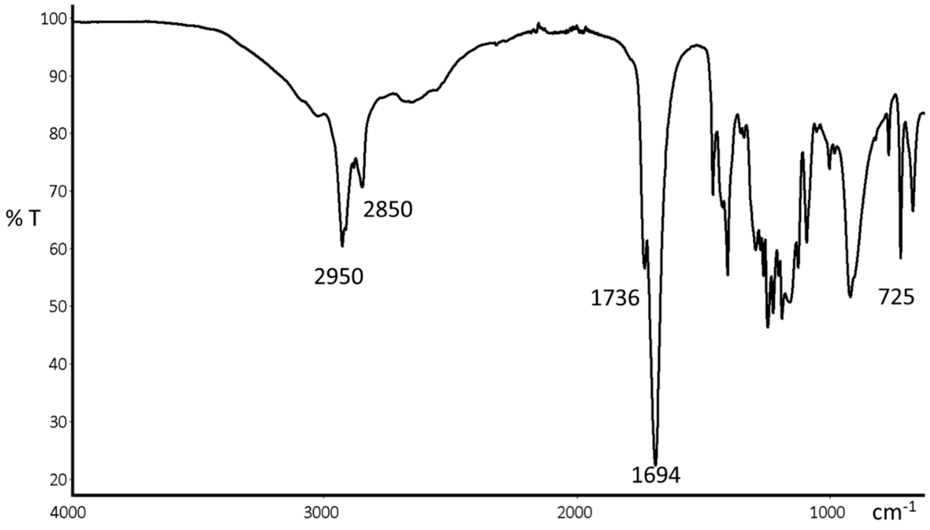

(**a**)

Figure S10. *Cont.*

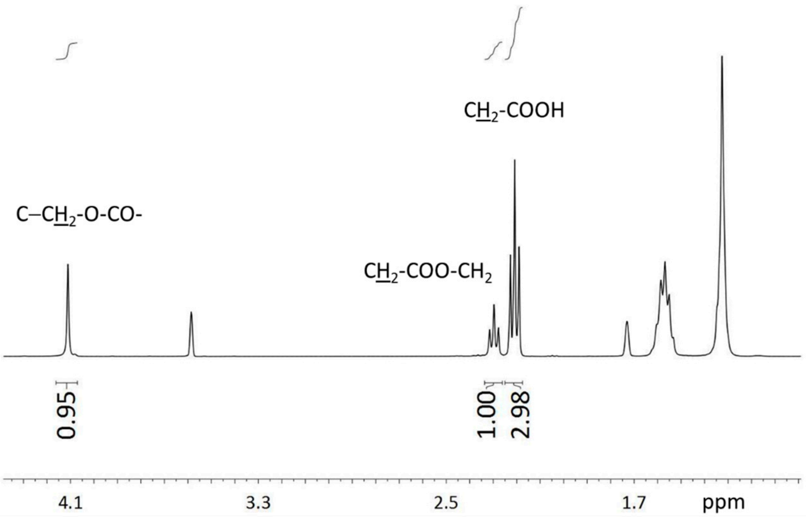

(**b**)

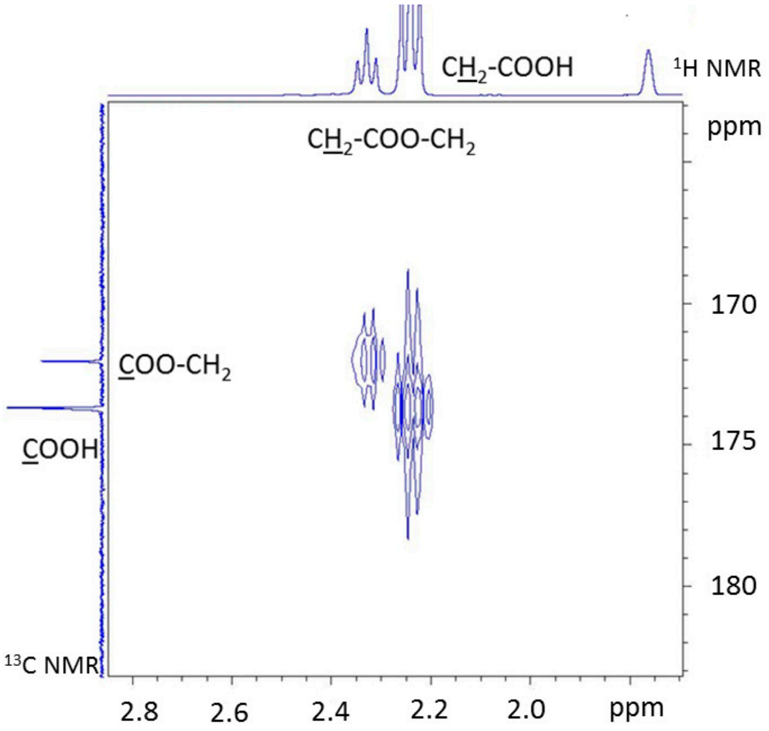

(**c**)

Figure S10. (**a**) IR spectrum of the pentaerythritol tetraazelate mixture; (**b**) ^{1}H-NMR spectrum of the pentaerythritol tetraazelate mixture; (**c**) Expanded HMBC spectrum of the pentaerythritol tetraazelate mixture showing correlations between two types of carbonyl groups and their adjacent methylene groups.

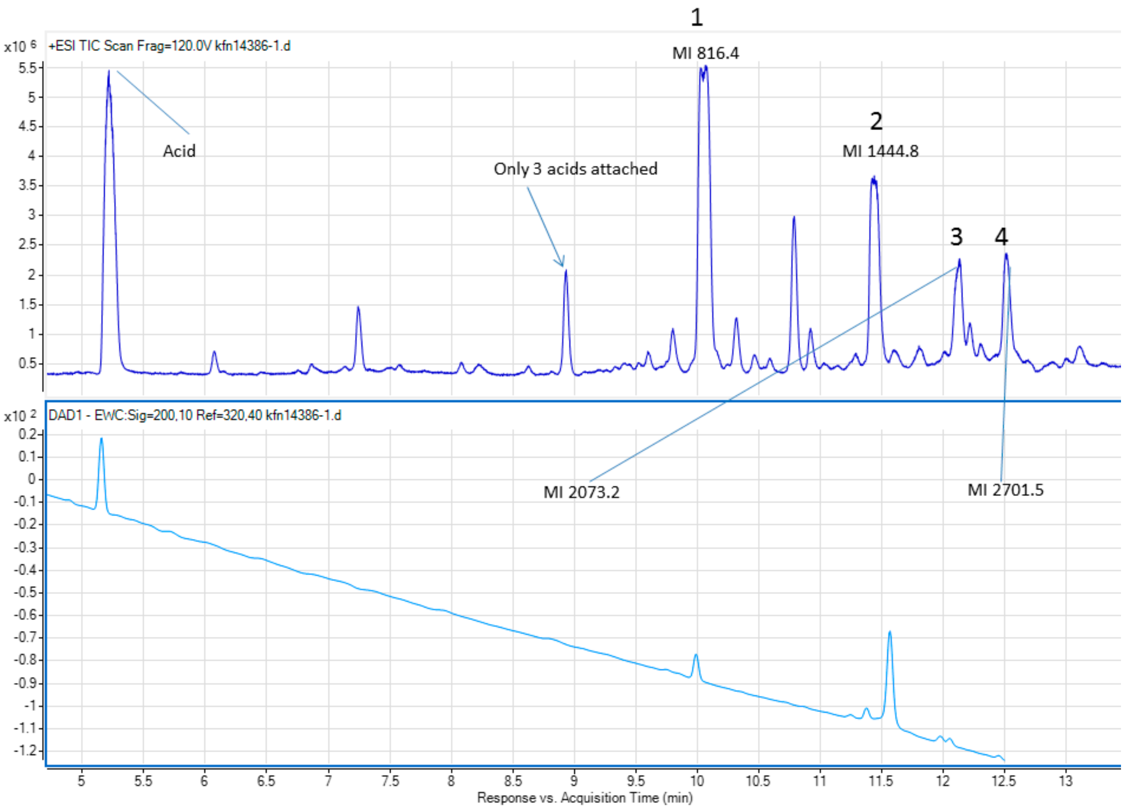

Figure S11. LC-MS analysis of the crude reaction mixture, where the **top** graphic shows the MS detector (ESI) and the **bottom** graphic shows the diode array detector (DAD).

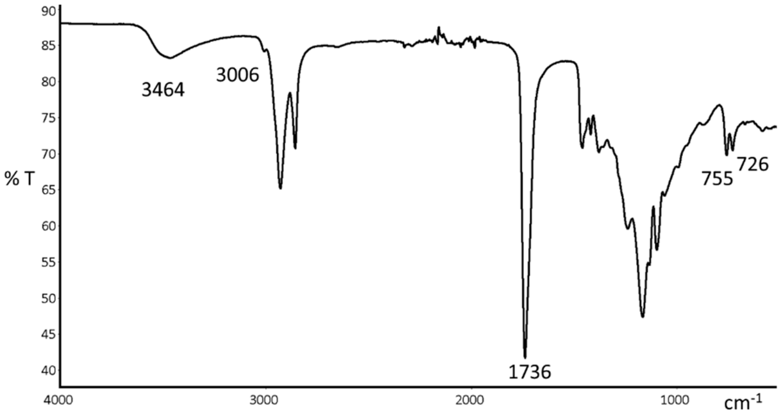

Figure S12. IR spectrum of UBP7.

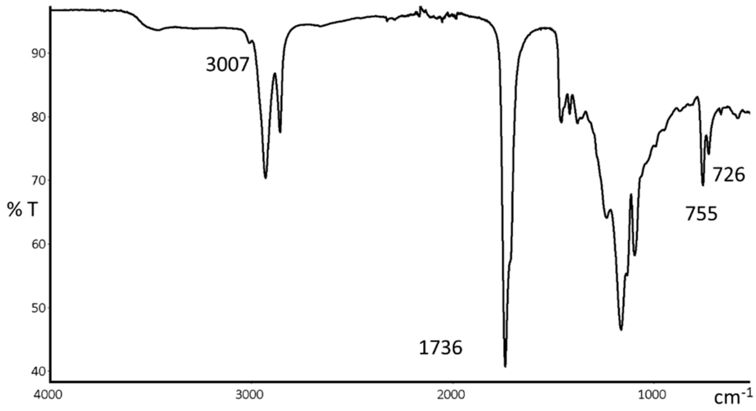

Figure S13. Expanded IR spectrum of UBP10.

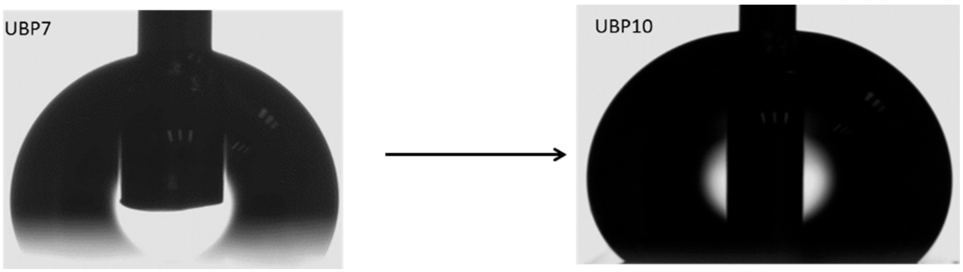

Figure S14. Increasing of WCA with decreasing glycerol content.

UBP 1

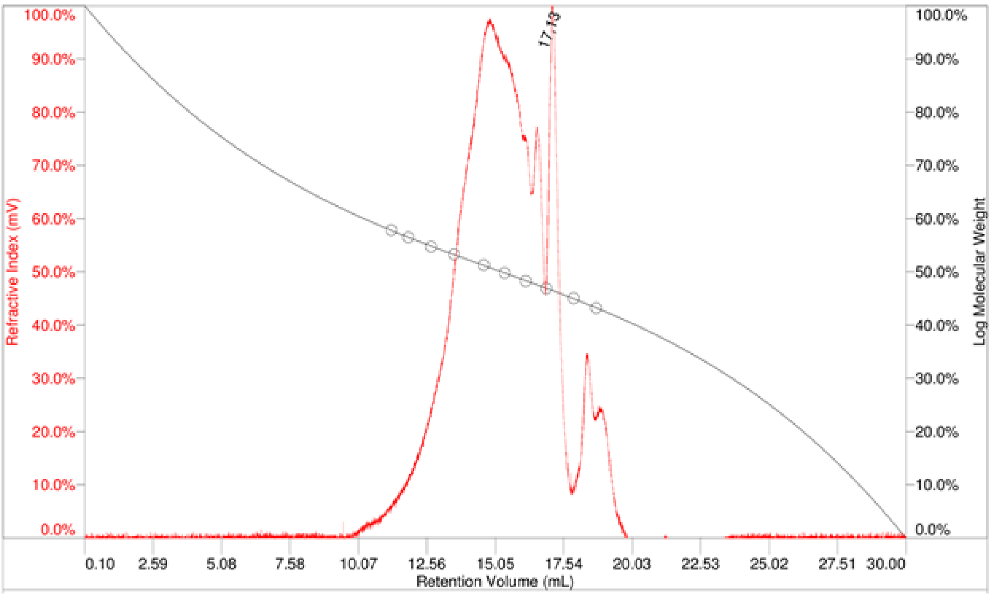

Figure S15. SEC chromatograph and data table of UBP1.

UBP 2

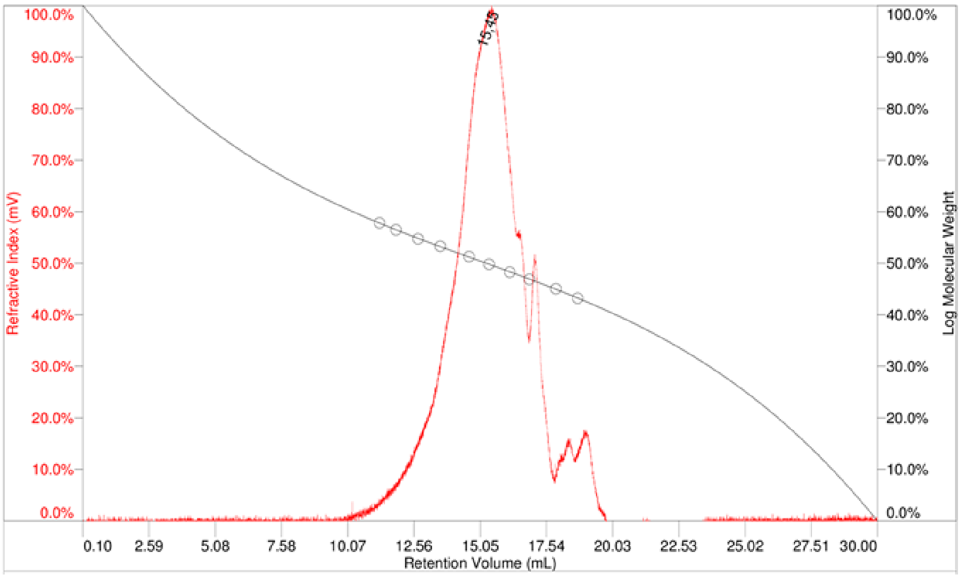

Figure S16. SEC chromatograph and data table of UBP2.

UBP 3

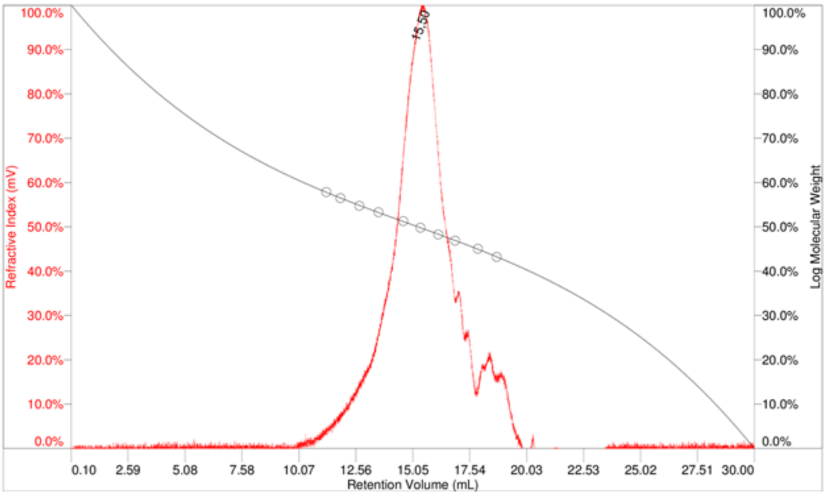

Figure S17. SEC chromatograph and data table of UBP3.

UBP 4

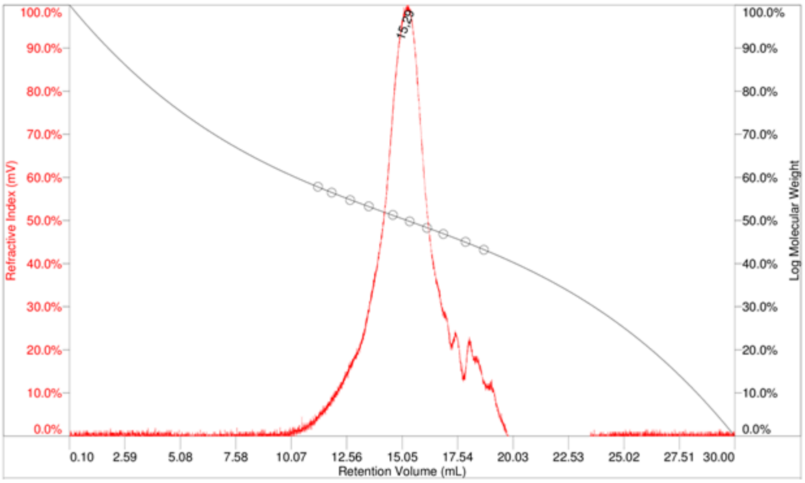

Figure S18. SEC chromatograph and data table of UBP4.

UBP 5

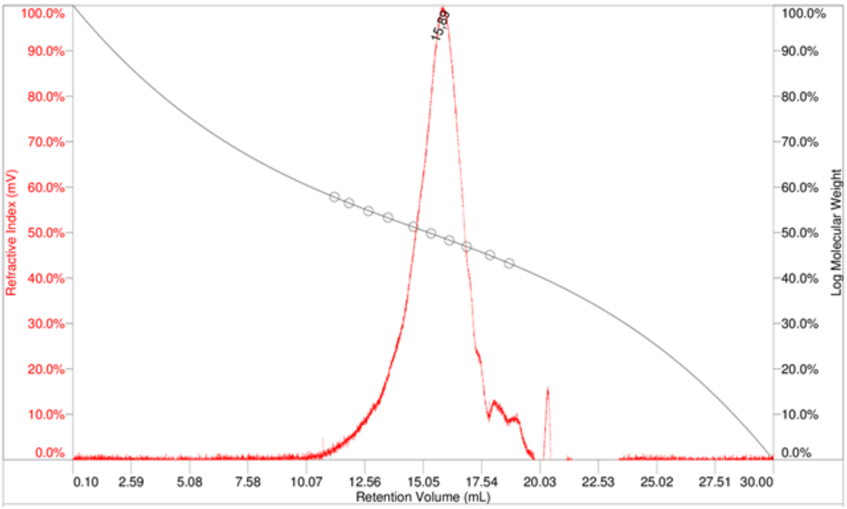

Figure S19. SEC chromatograph and data table of UBP5.

UBP 6

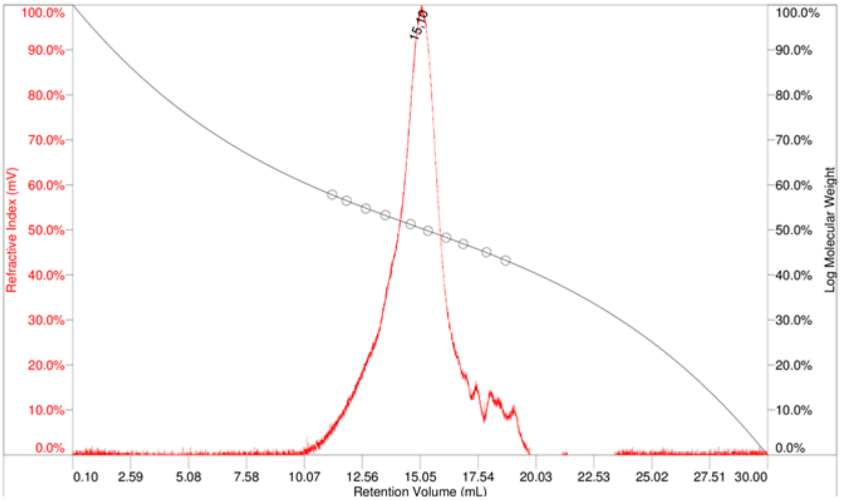

Figure S20. SEC chromatograph and data table of UBP6.

UBP 7

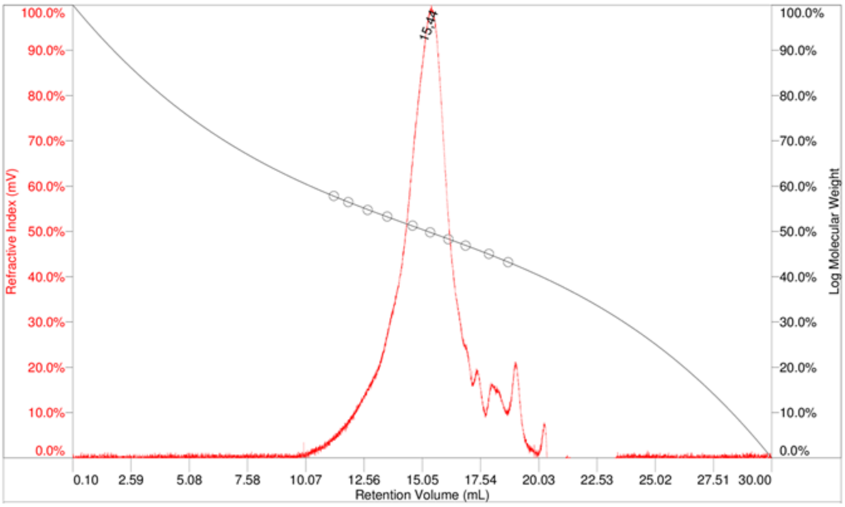

Figure S21. SEC chromatograph and data table of UBP7.

UBP 8

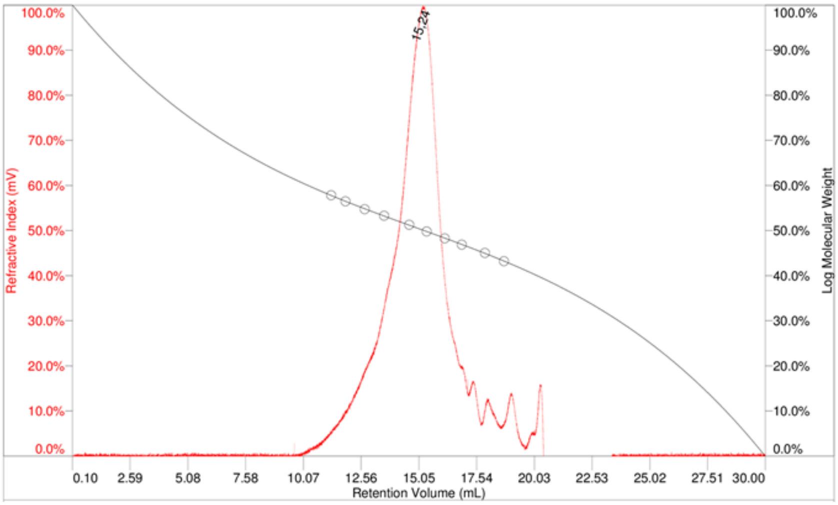

Figure S22. SEC chromatograph and data table of UBP8.

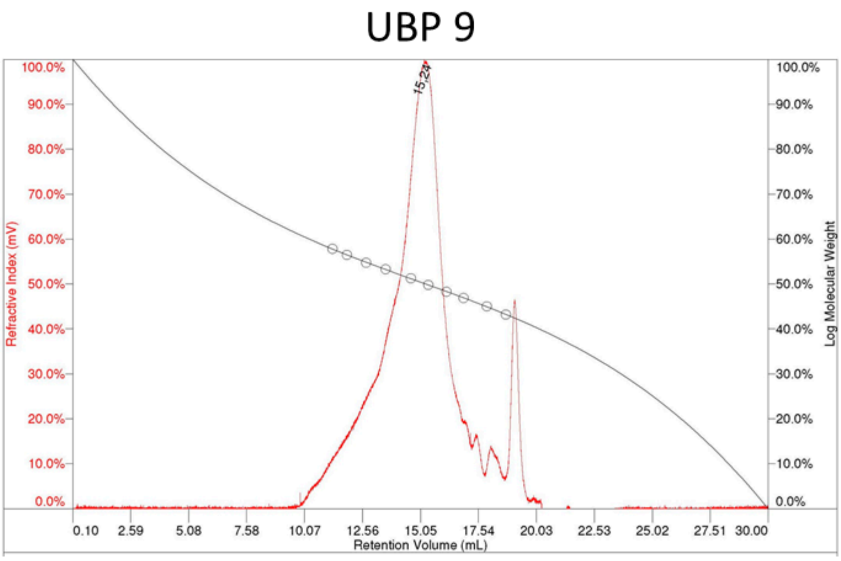

Figure S23. SEC chromatograph and data table of UBP9.

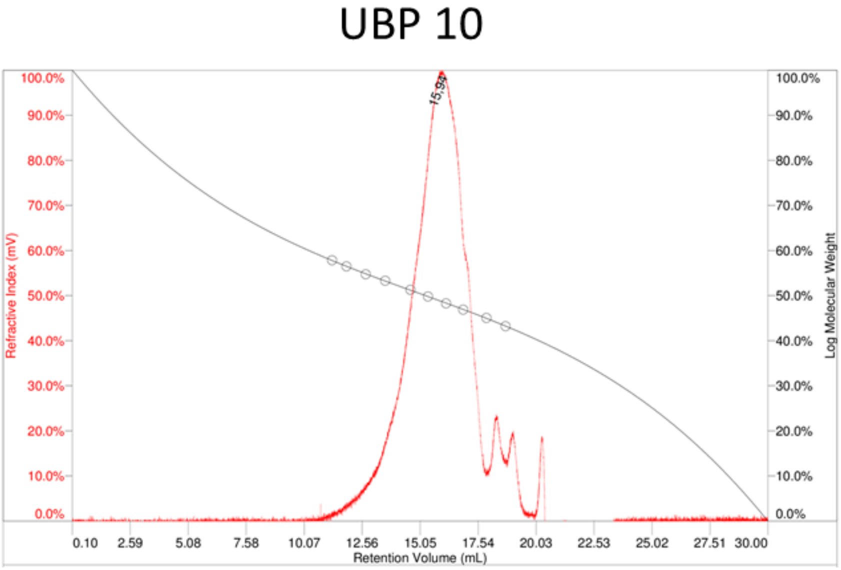

Figure S24. SEC chromatograph and data table of UBP10.

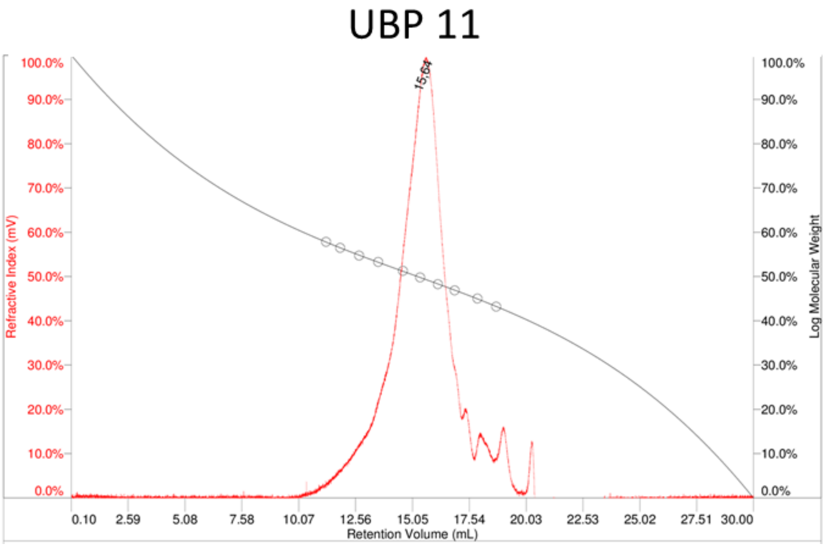

Figure S25. SEC chromatograph and data table of UBP11.
